# Survival gains needed to offset persistent adverse treatment effects in localised prostate cancer

**DOI:** 10.1038/bjc.2011.552

**Published:** 2012-01-24

**Authors:** M T King, R Viney, D P Smith, I Hossain, D Street, E Savage, S Fowler, M P Berry, M Stockler, P Cozzi, P Stricker, J Ward, B K Armstrong

**Affiliations:** 1 Psycho-oncology Co-operative Research Group (PoCoG), School of Psychology, University of Sydney, Room 148, Transient Building (F12), Sydney, NSW 2006, Australia; 2 Centre for Health Economics Research and Evaluation, University of Technology Sydney, Sydney, NSW, Australia; 3 Cancer Council NSW, Sydney, NSW, Australia; 4 Department of Finance & Economics, College of Business & Economics, Qatar University, Doha, Qatar; 5 Australian School of Advanced Medicine, Macquarie University Hospital and Clinic, Sydney, NSW, Australia; 6 Sydney Cancer Centre, Sydney, NSW, Australia; 7 NHMRC Clinical Trials Centre, University of Sydney, Sydney, NSW, Australia; 8 School of Public Health, University of Sydney, Sydney, NSW, Australia; 9 University of New South Wales, Urology Sydney and Continuum Healthcare Group, Sydney, NSW, Australia; 10 St Vincents Clinic, Sydney, NSW, Australia; 11 Department of Epidemiology & Community Medicine, University of Ottawa, Ottawa, Ontario, Canada

**Keywords:** prostate cancer, preferences, trade-off, utility, survival benefit, quality of life

## Abstract

**Background:**

Men diagnosed with localised prostate cancer (LPC) face difficult choices between treatment options that can cause persistent problems with sexual, urinary and bowel function. Controlled trial evidence about the survival benefits of the full range of treatment alternatives is limited, and patients' views on the survival gains that might justify these problems have not been quantified.

**Methods:**

A discrete choice experiment (DCE) was administered in a random subsample (*n*=357, stratified by treatment) of a population-based sample (*n*=1381) of men, recurrence-free 3 years after diagnosis of LPC, and 65 age-matched controls (without prostate cancer). Survival gains needed to justify persistent problems were estimated by substituting side effect and survival parameters from the DCE into an equation for compensating variation (adapted from welfare economics).

**Results:**

Median (2.5, 97.5 centiles) survival benefits needed to justify severe erectile dysfunction and severe loss of libido were 4.0 (3.4, 4.6) and 5.0 (4.9, 5.2) months. These problems were common, particularly after androgen deprivation therapy (ADT): 40 and 41% overall (*n*=1381) and 88 and 78% in the ADT group (*n*=33). Urinary leakage (most prevalent after radical prostatectomy (*n*=839, mild 41%, severe 18%)) needed 4.2 (4.1, 4.3) and 27.7 (26.9, 28.5) months survival benefit, respectively. Mild bowel problems (most prevalent (30%) after external beam radiotherapy (*n*=106)) needed 6.2 (6.1, 6.4) months survival benefit.

**Conclusion:**

Emerging evidence about survival benefits can be assessed against these patient-based benchmarks. Considerable variation in trade-offs among individuals underlines the need to inform patients of long-term consequences and incorporate patient preferences into treatment decisions.

Men diagnosed with localised prostate cancer (LPC) face difficult treatment decisions. Although the eradication of cancer is a major issue for most men (Zeliadt *et al*, 2006), controlled trial evidence about the survival benefits of the full range of treatment alternatives is limited (Wilt *et al*, 2008) and likely to remain so for some time. Survival benefits are offset by treatment complications, including problems with sexual, urinary and bowel function (Potosky *et al*, 2004; Sanda *et al*, 2008; Parker *et al*, 2009; Smith *et al*, 2009), issues considered important by patients and their partners (Zeliadt *et al*, 2006; van Tol-Geerdink *et al*, 2006a). Limitations in the evidence and personalised trade-offs between quantity and quality of life mean that no one therapy can be considered the preferred treatment for all men (Wilt *et al*, 2008). Although optimal treatment is influenced by tumour and patient characteristics (Sommers *et al*, 2007), patient preference is also an important factor (National Health and Medical Research Council (NHMRC), 2003; National Comprehensive Cancer Network, 2004; Zeliadt *et al*, 2006; Sommers *et al*, 2007). Many patients would like to be involved in decision making (Davison *et al*, 2004; van Tol-Geerdink *et al*, 2006b), but the complexity and uncertainty of the information required are major barriers. Physicians often bear the responsibility of assessing the options on the patient's behalf (Elstein *et al*, 2005), yet they are poor judges of patients' preferences (Elstein *et al*, 2004; Stalmeier *et al*, 2007).

Studies of patient preferences for treatment of LPC fall into three classes: those which examine actual treatment choices ('revealed preferences'); those which estimate the value ('utility') of various health states; and clinical decision analyses. Zeliadt *et al* (2006) review the former class, whereas Bremner *et al* (2007) review the latter two. Although the former help understand men's decision making processes, they provide limited insight into the relative tolerability of side-effects because typically the information presented to men at the point of decision-making is not standardised. Studies in the latter classes typically quantify preferences in terms of utility decrements caused by specific adverse effects (Lubeck *et al*, 2002; Bremner *et al*, 2007). Utility has traditionally been estimated with time trade off and standard gamble methods, and more recently with discrete choice experiments (DCE) (Ryan, 2004). When hypothetical health states are included in these 'stated preference' methods, they yield more accurate and valid results than those based on personalised health states because the description of health states is standardised (Chapman *et al*, 1998).

To date, no studies have explicitly expressed patient preferences for treatment of LPC in terms of the relative tolerability of adverse treatment-related effects or the survival gains needed to make persistent adverse effects worthwhile. Each of these is relevant to clinicians counselling patients on treatment decisions, and the latter perspective provides a benchmark against which emerging evidence about actual survival benefits of alternative treatments can be assessed. The aim of this study was to quantify these issues using a DCE.

## Materials and methods

### Study design and sample

**Figure 1:**
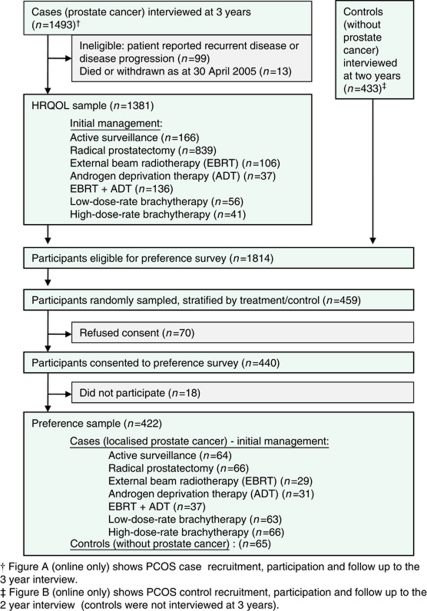
Flow diagram showing derivation of HRQOL and preference survey samples of the PCOS.

Data were obtained from the New South Wales (NSW) Prostate Cancer Care and Outcomes Study (PCOS). PCOS is a population-based (NSW, Australia) cohort study of men aged <70 when diagnosed with prostate cancer and recruited from the NSW Central Cancer Registry, and age and postcode matched controls (without prostate cancer) recruited via the White Pages telephone directory. Details of recruitment to PCOS and detailed demographic and clinical profiles of each treatment group and the controls are reported elsewhere (Smith *et al*, 2009). Flow diagrams for PCOS case and control recruitment and follow-up are given in Supplementary Figures A and B. Sample flow relevant to this paper is depicted in Figure 1. The study was approved by the ethics committees of the Cancer Council NSW, Cancer Institute NSW and NSW Department of Health. Informed consent was obtained from each participant.

### Preference elicitation method

A DCE was used to determine the utility of hypothetical health states that might result from treatment for LPC (Louviere *et al*, 2003). Each state was described in terms of 'attributes': treatment-related adverse effects and survival. We reviewed the literature and consulted with clinicians and a consumer representative to identify seven common treatment-related adverse effects; each was assigned three levels (Table 1). We also included average life expectancy and its uncertainty, with levels 4, 8 or 12 years and ±25%, 50% or 75%, respectively.

### Experimental design and sample size

Health states were constructed according to an experimental design. The 'full factorial' design contains all possible combinations of attributes and levels (3^9^=19 683 states). A statistically optimal subsample of 108 health states was selected (Street *et al*, 2005), arranged into 54 pairs ('choice sets') (Supplementary Figure C), then split into three versions (18 choice sets each). Twenty respondents per treatment group per version were needed (Louviere *et al*, 2000), thus 420 participants were required for the preference survey.

### Data collection

The preference survey was piloted (see technical Supplementary Appendix for details). Preference survey participants were randomly assigned to questionnaire version within treatment strata. Preference surveys were posted to subjects, who were then contacted by telephone and the data collected by telephone interview. Collection of the health-related quality of life (HRQOL) data is described elsewhere (Smith *et al*, 2009).

### Analysis of the HRQOL data

Prevalence of urinary, sexual and bowel problems, fatigue and other hormonal effects were estimated from the 3-year post-diagnosis HRQOL data. Seven questions from the long-form University of California Los Angeles Prostate Cancer Index (Litwin *et al*, 1998) that most closely corresponded to the seven attributes in the preference survey were used to classify each individual (*n*=1381) into one of the three categories (none, mild and severe) for each treatment-related adverse effect (see technical Supplementary Appendix for details).

### Analysis of the preference data

Preference data were analysed with random parameter logit models (McFadden and Train, 2000; Train, 2003) (see technical Supplementary Appendix for details). In summary, the utility impact of each treatment-related adverse effect level and survival was inferred from respondents' choices over the 54 pairs of hypothetical health states. Two sets of coefficients were estimated, one representing the mean utility impact of the attributes (fixed effects), the other representing variation among individuals in their preferences (random effects).

Relative tolerability of treatment-related adverse effects was addressed by ranking the fixed effects coefficients: those with the largest negative coefficients (greatest negative impact on utility) were interpreted as least tolerable, and those with the smallest negative coefficients or positive coefficients were interpreted as the most tolerable. Confidence intervals on coefficients were taken into account in interpreting the relative rankings.

The degree of variation among men in preferences was quantified by the distribution of the random effect coefficients. These are assumed to be normally distributed, so the majority (68%) lie within one s.d. of the fixed effect coefficients. As this represents the typical range of individual utility coefficients, it is a clinically relevant expression of preference heterogeneity.

The effect of treatment group on preferences was evaluated by estimating separate fixed effects models for each treatment group. As parameter estimates in discrete outcome models are confounded by error variance (Swait and Louviere, 1993), parameter estimates were not directly comparable across treatment groups. Rank ordering of parameter estimates was used to assess the similarity of preferences across the treatment groups, again taking confidence intervals into consideration. The effect of age on preferences for sexual function was addressed with a likelihood ratio test for the interaction of age (dichotomised at 65) with severe erectile dysfunction and severe loss of libido (see technical Supplementary Appendix for details).

### Estimation of survival gains needed

To estimate the survival gains needed to justify persistent treatment-related adverse effects, the value of changes in health utility associated with each level of each side effect was expressed in terms of survival time (see technical Supplementary Appendix for details). In summary, the survival gain needed to justify a chronic treatment-related adverse effect(s) was such that the value of this health state for this extended time (*T+*survival gain needed) was equivalent to the value of the base case (health state without treatment) for a survival time of 12 years (*T*). The base case was determined from the 3-year post-diagnosis HRQOL data of men initially managed with active surveillance; in the absence of a randomised design, this group was deemed the most valid comparator. The survival gains needed for the additional burden of each persistent treatment-related adverse effect level singly and for each commonly occurring combination of effects were calculated, and their distributions were simulated. We report medians and 2.5 and 97.5 centiles, analogous to 95% confidence intervals estimated by bootstrap methods.

## Results

### Study participants

Table 2 shows the characteristics of the 1381 PCOS cases with complete 3-year HRQOL data (the 'HRQOL sample') and the 422 men, who participated in the preference survey (the 'preference sample'). Supplementary Figures A and B and Figure 1 show the derivation of these samples.

**Figure 2:**
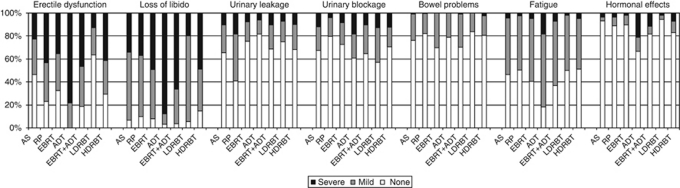
Prevalence of urinary, sexual and bowel problems, fatigue and other hormonal effects 3 years after diagnosis by treatment group (HRQOL sample, *n*=1381): active surveillance (AS, *n*=166), radical prostatectomy (RP, *n*=839), external beam radiotherapy (EBRT, *n*=106), ADT (*n*=37), combined therapy (EBRT+ADT, *n*=136), low-dose rate brachytherapy (LDRBT, *n*=56), high-dose rate brachytherapy (HDRBT, *n*=41). White=none, grey=mild, black=severe.

Prevalence of adverse effects (Table 1) is given by treatment group in Figure 2. Only 1.2% of the HRQOL sample (including 1.2% of the active surveillance group) reported none of the seven side-effects/symptoms 3 years after diagnosis. Prevalence of adverse effects for the 357 cases in the preference sample in also given Table 1. Severe erectile dysfunction and severe loss of libido were common in both the HRQOL sample and the DCE subsample, slightly moreso in the latter because of stratified sampling by treatment.

Relative tolerability and preference heterogeneity are given in Table 3. The rank order of treatment-related adverse effects was similar across treatment groups (Supplementary Table A). Severe urinary leakage, urinary blockage and bowel symptoms were the three least tolerable side effects in five of the six treatment groups, and in the active surveillance and control groups. There was no effect of age on preferences: all interaction terms were non-significant (*P*>0.05), other estimates remained similar, and the function value improved very little (−2 log likelihood=3.39, df=3, one-sided *P*=0.34).

**Figure 3:**
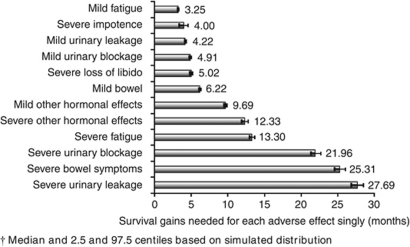
Additional months of life needed^†^ to compensate for each persistent treatment-related adverse effect in excess of a base case of mild loss of libido with no other problems and 12 year life expectancy.

The majority (59%) of men initially managed with active surveillance reported mild loss of libido at 3 years (Figure 2); this was used as the base case. Additional survival needed to compensate for persistent treatment-related adverse effect ranged from about 2 years for the severe levels of the three least tolerable treatment-related adverse effects (both aspects of urinary function and bowel problems), through to about 1 year for severe fatigue and other hormonal effects, about 10 months for mild other hormonal effects and 3–6 months for the remainder (Figure 3).

Many men reported more than one adverse effect at 3 years. There were 3^7^=2187 unique health states described by the three levels of the seven adverse effects. It was not practicable to estimate survival gains for each one. As the function used to calculate survival gains needed (expression 4 in the technical Supplementary Appendix) is not linear, the cumulative effect of multiple adverse effects cannot be derived simply by summing over component effects. We therefore simulated the survival gains needed for the three most common treatment-related adverse effect profiles for each treatment group (Supplementary Table B). Approximations based on the simple sum of the component adverse effects in most cases fell within the 2.5 and 97.5 centiles of the simulated distributions. For example, severe erectile dysfunction alone would be compensated by a 4-month survival benefit and severe libido loss with 5.02 months (Figure 3), giving a sum of 9.02, which is a reasonable approximation of the simulated estimate of 9.14 months, well within the 95% confidence interval (8.46–9.80, Supplementary Table B).

## Discussion

Severe urinary dysfunction and bowel symptoms were least tolerable, severe hormonal effects and fatigue were somewhat more tolerable, and severe sexual dysfunction was relatively benign, having about the same negative impact as mild urinary dysfunction and bowel symptoms. Other hormonal effects were the least tolerable of the mild treatment-related adverse effects. These patterns were consistent across treatment groups and unaffected by age. Overlaid on these aggregate results was substantial variation in individual preferences, consistent with the decision analysis of Sommers *et al* (2007) and two preference studies (Singer *et al*, 1991; Stewart *et al*, 2005). Ours is the first study to quantify this variation. This leads to two important observations. First, virtually all men were averse to severe urinary leakage (predominantly negative individual coefficients), although some men's aversion was much greater than others'. The same was true for all severe adverse effects except severe loss of libido (where the utility impact was consistently small across respondents). Second, the lower end of the range of individual utility decrements for severe urinary leakage, severe bowel symptoms and severe urinary blockage was typically more negative than the upper end of the range of decrements for most of the mild side effects (MUI±s.d. in Table 3). Together, these two observations have an important clinical implication: patients are likely to make decisions about treatment based on severe adverse effects, not mild ones.

Men managed with active surveillance commonly experienced mild loss of libido 3 years after diagnosis; this formed the base case for estimating survival benefit. Severe erectile dysfunction and severe loss of libido were prevalent, particularly in men who had androgen deprivation therapy (ADT); 4 and 5 months additional survival, respectively, were needed to justify these singly and about 9 months if experienced together. Urinary leakage was most prevalent in the radical prostatectomy group; 4 months additional survival was needed for mild levels and about 28 months for severe levels. Mild bowel problems were most prevalent in men who had external beam radiotherapy; about 6 months was needed to justify these. Of the 18 most common health states 3-years post-diagnosis, 10 required <6 months of additional survival; the 4 health states most common after radical prostatectomy were among these. For 6 of the 18 most common health states, survival benefits needed ranged from 1–3 years; of these, 5 were relatively common in the ADT group, accounting for 33.5% of that group in total. All involved severe libido loss and severe erectile dysfunction, accounting for about 9 months of the survival benefit needed.

Thus we found that relatively modest survival benefits were sufficient to offset the most common side effects of treatments for prostate cancer for about two-thirds of the most common health states 3-years post-diagnosis. These are similar to those judged by women with early breast cancer as sufficient to make adjuvant chemotherapy or endocrine therapy worthwhile (Duric and Stockler, 2001; Thewes *et al*, 2005). However, even substantial survival benefits were insufficient to offset severe urinary dysfunction, which is not rare (reported by 14% of our HRQOL sample at 3 years, with similar prevalence estimates in other population-based studies (Potosky *et al*, 2000, 2004; Sanda *et al*, 2008; Gore *et al*, 2009; Mols *et al*, 2009; Smith *et al*, 2009)). Men considering their options should be counselled about the risks of treatment-related adverse effects, their likely effects on quality of life, and possible remedial measures.

The results of our study corroborate the findings of many utility studies in LPC: mild symptoms have higher utility than severe, and urinary and bowel symptoms have much lower utility than sexual dysfunction and loss of libido (Bremner *et al*, 2007). Our study adds to this literature by providing estimates of survival gains required for each common adverse treatment effect, thereby translating utilities into something practical for clinicians and patients–survival gains needed to justify persistent problems. By incorporating the HRQOL data from the our larger PCOS study, the current paper uniquely reveals the potential overall impact of the trade-offs and survival gains required for adverse treatment side effects.

Although there are numerous studies of patient preferences in LPC prostate cancer, ours provides the most comprehensive set of treatment-related adverse effects. Our population-based sample is a major strength, containing experiences of all treatment options and controls. Like many previous studies, ours was retrospective, with men bringing personal experience to their hypothetical choices. We avoided bias arising from patient's preconceptions by not associating health states with treatments (Zeliadt *et al*, 2006). Although real choices made before treatment may differ from hypothetical choices made with the benefit of hindsight, arguably the latter are more informative than the former for future men facing similar decisions. Our controls were men of similar age and demographic profile to our cases, and therefore most like men facing a primary treatment decision. It is noteworthy that their results were similar to those of each of the treatment groups (as shown in Supplementary Table A), suggesting that men's preferences are infact not markedly affected by experience of treatment. We limited our sample of cases for the DCE to men with localised disease as we felt their opinions were most informative for men facing future choices about curative treatments for LPC. The attitudes of patients whose disease progressed, particularly those who died early because of very aggressive disease, have not been captured in our study, and so our results may not generalise to this group. Whether such patients would have preferred to be alive even with severe adverse effects is a question that is beyond the scope of this study.

Previous studies have various limitations: considering only one/some adverse effects (Singer *et al*, 1991; Saigal *et al*, 2001; Bruner *et al*, 2004); assigning different severity levels to adverse effects (Saigal *et al*, 2001; Sculpher *et al*, 2004); assessing only one or two treatment options (Singer *et al*, 1991; Bruner *et al*, 2004; Sculpher *et al*, 2004; Jenkins *et al*, 2005); sampling respondents from only one treatment type (Smith *et al*, 2002;Bruner *et al*, 2004); combining bladder and bowel problems into one attribute (Chapman *et al*, 1999; Knight *et al*, 2004); investigating just a few hypothetical health states (Chapman *et al*, 1999; Saigal *et al*, 2001; Bruner *et al*, 2004; Knight *et al*, 2004; Jenkins *et al*, 2005); using patients' rating of their own health states (Smith *et al*, 2002; Krahn *et al*, 2003). These factors limit the comparability of our results for relative tolerability of adverse effects with those from other studies (Lubeck *et al*, 2002; Bremner *et al*, 2007). The best comparison is with the pooled estimates from a meta-analysis (Bremner *et al*, 2007); taking severity into account, the ordering of sexual, urinary and bowel dysfunction was as we observed. This ranking is consistent with the observation that sexual dysfunction (although relatively common) is not always correlated with sexual bother, whereas poorer urinary and bowel function (although relatively rare) are generally highly correlated with greater bother (Reeve *et al*, 2006; Smith *et al*, 2009). The relative tolerability of these adverse effects in this patient population may be influenced by baseline prevalence. In particular, high prevalence of mild and severe erectile dysfunction and loss of libido at baseline may contribute to these attributes being assessed as more tolerable than less commonly experienced adverse effects such as severe urinary and bowel problems.

To our knowledge, only three studies provide explicit estimates of survival trade-offs for treatment for LPC. In one, men with locally advanced prostate cancer were asked to make hypothetical choices between short- and long-term ADT, involving the trade-off of survival against sexual potency, hot flashes, fatigue and osteoporosis (Wilke *et al*, 2010). On average, these men were willing to trade 8% of a 5-year survival (4.8 months) for the better sexual potency and sexual drive provided by the short- versus the long-term ADT. In the second study, using a similar method but involving only sexual potency and survival, men without prostate cancer were willing to trade 10% of a 5-year survival (about 6 months) to maintain sexual potency (Singer *et al*, 1991). The results from these two studies are not dissimilar to our estimates of 3 months of a 12-year life expectancy for mild erectile dysfunction and 4 months for severe erectile dysfunction. The third study used a DCE comprising six common adverse effects of ADT (hot flushes, breast swelling or tenderness, physical energy, sex drive, ability to maintain an erection and diarrhea; some with two levels, others with three) and life expectancy (Sculpher *et al*, 2004). Survival trade-offs were estimated by marginal rates of substitution (MRS) between life expectancy and other attributes, showing for example that 1.8 months of additional life expectancy was needed to move from moderate to mild levels of diarrhoea or from mild to absent. By contrast we found about 6 months was required to compensate for occasional loose bowel movements. All of Sculpher *et al*'s (2004) survival trade-off estimates were two to four times smaller than ours. This is not because we used compensating variation (CV) rather than MRS; MRS calculated from our data were 1.5 to 2 times larger than our CV estimates, widening the difference with Sculpher *et al*'s (2004). We suspect the main cause of difference in our estimates arises from the way survival gain is expressed. The life expectancy attribute in Sculpher *et al*'s (2004) DCE had only two levels (2 and 4 months, framed as additional survival without specifying the average life expectancy against which this gain is assessed). Our life expectancy attribute had three levels (4, 8 and 12 years, framed as life expectancy, thus any calculated gain in life expectancy is interpreted relative to this average). Despite this large difference in time scale, the coefficients are not dissimilar: 0.23 (Sculpher *et al*, 2004) versus 0.34 (ours). Our survival benefit time scale was realistic in terms of life expectancy of participants. Our CV approach has several advantages: it allows for combinations of adverse effects (more clinically relevant than single side effects); it takes into account an empirically based base case and it quantifies additional survival required to compensate for loss in quality of life because of treatment (again with empirically based health states).

We found that mild erectile dysfunction had a significant positive utility value (0.14, Table 3). This was probably because of the high prevalence of severe erectile dysfunction in the DCE sample (44%, Table 1). Although the positive utility impact of mild erectile dysfunction can be explained, it can become problematic when added to other negative utility effects. For example, our results paradoxically suggest that severe libido loss requires 5.02 months survival gain, whereas severe libido loss plus mild erectile dysfunction requires only 1.82 months. This empirical quirk should be kept in mind when interpreting results in Supplementary Table B for heath states including mild erectile dysfunction. It also highlights the potential gains in utility of remedial measures for erectile dysfunction.

Increasing uptake of prostate-specific antigen testing and attendant risks of overdiagnosis and overtreatment present clinical and ethical dilemmas (Barry, 2009). If treated, men are at risk of adverse treatment-related effects and a contestable survival benefit (Potosky *et al*, 2004; Sanda *et al*, 2008; Gore *et al*, 2009; Mols *et al*, 2009; Smith *et al*, 2009). The most fruitful avenues for future research are therefore those that increase our understanding of variation in individual preferences, the relationship of preferences to treatment decisions and the effectiveness of different ways of presenting complex choice information to men before treatment decisions are made, of treatment innovations to minimise treatment-related adverse effects and of early interventions to ameliorate them. The ultimate goal is evidence-based shared decision making that matches management and outcomes to patients' preferences, thereby maximising patient utility after diagnosis of LPC.

## Figures and Tables

**Table 1:** Treatment-related adverse effects included as attributes in the preference survey and their prevalence at 3-year follow-up in 1381 PCOS participants who had localised prostate cancer at diagnosis and no recurrence at 3 years, and in the subset of 357 men (PCOS cases) who participated in the DCE

**Attribute**	**Level**	**Wording**	**PCOS case participants (*n*=1381) (%)**	**DCE case participants (*n*=357) (%)**
Erectile dysfunction	Base	No problems achieving an erection when you want one	27	27
	Mild	Some problems achieving an erection when you want one	33	29
	Severe	Never able to achieve an erection when you want one	40	44
				
Loss of libido	Base	No change in sexual desire	8	7
	Mild	Less sexual desire	50	43
	Severe	Complete loss of sexual desire	41	51
				
Urinary leakage	Base	No problems with leaking urine	53	64
	Mild	Occasional problems with leaking urine	33	23
	Severe	Severe problems with leaking urine (no urinary control whatsoever)	14	12
				
Urinary blockage	Base	No problems with urine blockage	74	67
	Mild	Some problems with urine blockage (have a weak urine stream but get some relief or comfort afterwards)	19	23
	Severe	Severe problems with urine blockage (continually feeling the need to urinate but passing very little with no relief afterwards)	7	10
				
Bowel symptoms	Base	No bowel problems	79	75
	Mild	Occasional loose bowel movements with discomfort/pain	20	25
	Severe	Very frequent loose bowel movements with discomfort/pain and leakage	1	1
				
Fatigue	Base	No change in your energy level	47	44
	Mild	Some tiredness and loss of energy	49	51
	Severe	Severe tiredness and loss of energy	4	5
				
Hormonal effects	Base	No hot flushes or moodiness	88	85
	Mild	Mild hot flushes and moodiness	7	7
	Severe	Severe hot flushes and moodiness	5	8

Abbreviation: PCOS=Prostate Cancer Outcomes Study.

**Table 2:** Characteristics of PCOS case participants who contributed HRQOL data at 3-year follow-up (*n*=1381) and PCOS cases and controls who completed the preference survey (*n*=422)

	**HRQOL sample *n* (% of 1381)**	**Preference sample *n* (% of 422)**
*Numbers per treatment strata*		
Cases (localised prostate cancer)		
Radical prostatectomy	839 (46.3)	64 (15.2)
External beam radiotherapy (EBRT) only	106 (5.8)	66 (15.6)
Androgen deprivation therapy (ADT) only	37 (2.0)	37 (8.8)
Combined EBRT and ADT	136 (7.5)	64 (15.2)
Low-dose rate brachytherapy	56 (3.1)	31 (7.3)
High-dose rate brachytherapy	41 (2.3)	29 (6.9)
Active surveillance	166 (9.2)	66 (15.6)
Controls (without prostate cancer)		65 (15.4)
		
*Age-group at 3-year follow-up or preference survey*		
<55	64 (4.6)	21 (5.0)
55–59	186 (13.5)	39 (9.2)
60–64	342 (24.8)	84 (19.9)
65–69	442 (32.0)	149 (35.3)
70–74	347 (25.1)	129 (30.6)
Mean (s.d.) age	65.0 (5.7)	65.9 (5.7)
		
*Highest level of education*		
Less than high school certificate	57 (4.1)	20 (4.7)
Completed high school	953 (69.0)	290 (68.7)
University or college degree	364 (26.4)	110 (26.1)
Unknown	7 (0.5)	2 (0.5)
		
*Marital status at diagnosis*		
Married or living as married	1160 (84.0)	350 (82.9)
Never married, divorced, separated or widowed	217 (15.7)	72 (17.1)
Missing	4 (0.3)	0 (0.0)
		
*Self reported health 3 year after diagnosis*		
Poor	66 (4.8)	11 (3.1)
Fair	176 (12.7)	55 (15.4)
Good	338 (24.5)	98 (27.5)
Very good	519 (37.6)	126 (35.3)
Excellent	275 (19.9)	64 (17.9)
Missing	7 (0.5)	3 (17.9)
		
*PSA level at diagnosis (ng ml* ^*−1*^ *)*		
<4.0	140 (10.1)	35 (9.8)
4.0–9.9	799 (57.9)	163 (45.7)
10.0–19.9	282 (20.4)	87 (24.4)
20+	129 (9.3)	65 (18.2)
Missing	31 (2.2)	7 (2.0)
		
*Gleason score at diagnosis*		
2–5	135 (9.8)	36 (10.1)
6	651 (47.1)	151 (42.3)
7	465 (33.7)	120 (33.6)
8–9	114 (8.3)	47 (13.2)
Missing	16 (1.2)	3 (0.8)
		
Median months between diagnosis and preferences survey (cases)		46 (32–60)
Median months between recruitment and preferences survey (controls)		30 (27–50)

Abbreviations: HRQOL=health-related quality of life; PCOS=Prostate Cancer Outcomes Study;PSA=prostate specific antigen.

**Table 3:** Relative tolerability of treatment-related adverse effects (mean utility impact) and preference variability (range of individual utility coefficients) based on estimates from the random parameter logit model

			**Range of individual coefficients^b^**		
**Preference survey attributes, ranked by impact in utility**	**Mean utility impact (MUI)^a^** **coefficient (95% CI)**	***P*** ^*^	**MUI±1 s.d.**		***P*** ^**^
Severe urinary leakage	−1.33 (−1.49, −1.16)	<0.001	−2.21	−0.44	<0.001
Severe bowel symptoms	−1.23 (−1.40, −1.07)	<0.001	−2.13	−0.33	<0.001
Severe urinary blockage	−1.08 (−1.22, −0.93)	<0.001	−1.73	−0.42	<0.001
Severe hormonal effects	−0.63 (−0.77, −0.49)	<0.001	−1.20	−0.05	<0.001
Severe fatigue	−0.62 (−0.73, −0.50)	<0.001	−0.95	−0.28	<0.001
Mild hormonal effects	−0.43 (−0.54, −0.31)	<0.001	−0.57	−0.28	0.24
Severe erectile dysfunction	−0.30 (−0.43, −0.16)	<0.001	−1.17	0.58	<0.001
Mild urinary blockage	−0.23 (−0.35, −0.12)	<0.001	−0.37	−0.09	0.40
Mild bowel symptoms	−0.27 (−0.38, −0.15)	<0.001	−0.32	−0.21	0.69
Severe libido loss	−0.21 (−0.32, −0.10)	<0.001	−0.27	−0.15	0.87
Mild urinary leakage	−0.20 (−0.31, −0.09)	0.001	−0.30	−0.09	0.56
Mild fatigue	−0.14 (−0.25, −0.03)	0.01	−0.18	−0.10	0.70
Mild erectile dysfunction	0.14 (0.02, 0.25)	0.025	0.08	0.19	0.60
Mild libido loss	0.04 (−0.08, 0.15)	0.55	−0.15	0.20	0.13

^*^, ^**^P-value for test of null hypothesis that fixed (^*^) and random (^**^) parameter estimates are equal to zero.

a The mean (β̂_*k*_ in Equation 2 in Supplementary Appendix) reflects the impact on utility of each attribute on average across respondents.

b The degree of variation among men in preferences (preference heterogeneity) was quantified with the s.d. of the distribution of the 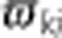 (see Equation 2 in technical Supplementary Appendix). Since the 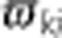 are normally distributed, the majority (68%) lie within one s.d. of the mean. This range therefore represents the typical range of individual utility coefficients.
