# Supplementary material for: Survival gains needed to offset persistent adverse treatment effects in localised prostate cancer
Source: Br J Cancer. 2012 Jan 24;106(4):638–45. doi: 10.1038/bjc.2011.552 (PMC3324299; doi:10.1038/bjc.2011.552)
Supplement: Supplementary Figure 1 [file bjc2011552x1.ppt]

## Slide 1
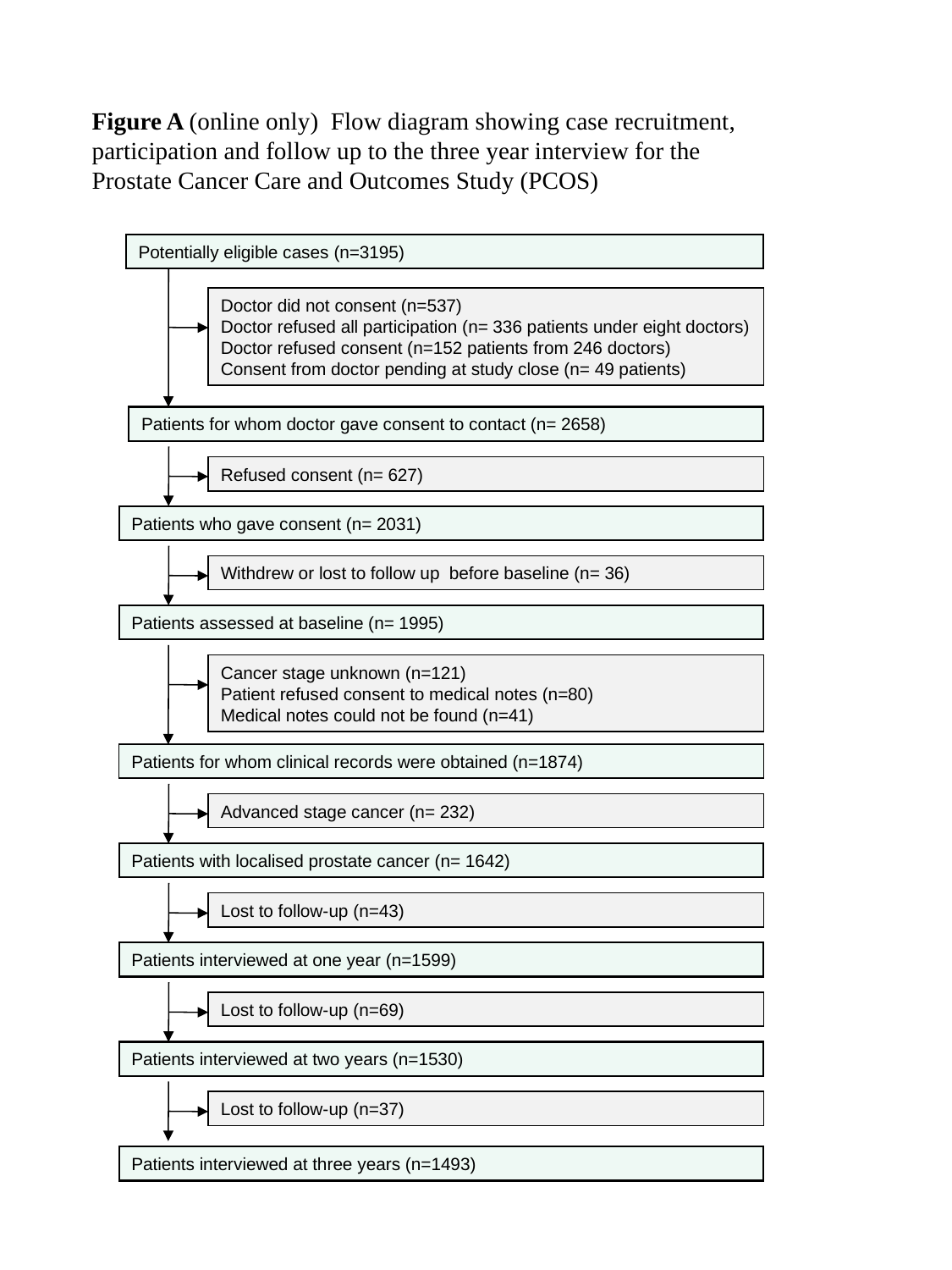

Figure A (online only) Flow diagram showing case recruitment, participation and follow up to the three year interview for the Prostate Cancer Care and Outcomes Study (PCOS)
Potentially eligible cases (n=3195)
Doctor did not consent (n=537)
Doctor refused all participation (n= 336 patients under eight doctors)
Doctor refused consent (n=152 patients from 246 doctors)
Consent from doctor pending at study close (n= 49 patients)
Patients for whom doctor gave consent to contact (n= 2658)
Refused consent (n= 627)
Patients who gave consent (n= 2031)
Withdrew or lost to follow up before baseline (n= 36)
Patients assessed at baseline (n= 1995)
Cancer stage unknown (n=121)
Patient refused consent to medical notes (n=80)
Medical notes could not be found (n=41)
Patients for whom clinical records were obtained (n=1874)
Advanced stage cancer (n= 232)
Patients with localised prostate cancer (n= 1642)
Lost to follow-up (n=43)
Patients interviewed at one year (n=1599)
Lost to follow-up (n=69)
Patients interviewed at two years (n=1530)
Lost to follow-up (n=37)
Patients interviewed at three years (n=1493)
