# Supplementary material for: Survival gains needed to offset persistent adverse treatment effects in localised prostate cancer
Source: Br J Cancer. 2012 Jan 24;106(4):638–45. doi: 10.1038/bjc.2011.552 (PMC3324299; doi:10.1038/bjc.2011.552)
Supplement: Supplementary Appendix [file bjc2011552x2.doc]

**ONLINE TECHNICAL APPENDIX**

***Prevalence of treatment-related adverse effects***

To estimate the prevalence of urinary, sexual and bowel problems, fatigue and other hormonal effects, the three year post-diagnosis HRQOL data were coded as follows. Seven questions from the long-form University of California Los Angeles Prostate Cancer Index (UCLA-PCI)[1] that most closely corresponded to the seven attributes in the preference survey were used to classify each individual (n= 1381) into one of three categories (none, mild, severe) for each treatment-related adverse effect.

| **Preference survey (DCE) attribute** | **UCLA-PCI question, included in PCOS HRQOL questionnaire battery** | **Coding of UCLA-PCI response options to match the three severity levels used in the DCE** | | |
| --- | --- | --- | --- | --- |
|  |  | **None** | **Mild** | **Severe** |
| Erectile function | How would you describe the usual quality of your erections in the last 4 weeks | Firm enough for intercourse | Not firm enough for intercourse or firm enough for masturbation and foreplay only | None |
| Libido/sexual desire | Your level of sexual desire | Very good | Fair, good | Very poor, poor |
| Urinary leakage | In the last 4 weeks, how often have you leaked urine | Not at all | About once a week, less than once a week | Every day |
| Urinary blockage | How big a problem was weak urine stream or incomplete emptying | No problem | A very small problem, a small problem | A moderate problem or severe problem |
| Bowel problems | How much distress did your bowel movements cause you in the last 4 weeks | None | Moderate or little | Severe |
| Tiredness or lack of energy | How often did you have a lack of energy | None of the time | A good bit, some or a little of the time | All of the time, most of the time |
| Hot flushes and moodiness | In the last four weeks, how often have you experienced hot flushes | Rarely or never | About once a week | Several times a day, about once a day, several times a week |

***Preference Survey Pilot***

Two survey formats were tested: 18 ‘choice sets’ of pairs of hypothetical treatment scenarios) and 9 choice sets of triples. Both formats were administered to six men who had been treated for prostate cancer. In general, they found the material comprehensible and the choice tasks in both designs were manageable, but the pair design was preferred. Completion times ranged from 15-45 minutes. Suggestions regarding rewording were incorporated. The questionnaire was then pilot tested on 25 PCOS participants (not preference survey participants) as a Computer Assisted Telephone Interview (CATI), and further minor modifications made.

***Statistical modelling of the preference data***

# The statistical modelling described below was used to address five specific objectives:

# To determine the impact of persistent treatment-related adverse effects on utility on average, and hence the relative tolerability of these effects;

# To determine the degree to which these impacts varied among individuals (preference heterogeneity);

# To determine the degree to which these impacts differed by treatment group;

# To determine whether attitudes towards loss of sexual function as a result of treatment differed by age; and

# To determine the survival gains needed for common persistent treatment-related adverse effects, singly and in combination.

**Random parameter logit model:** Under random utility theory [2], the utility that individual *i* derives from alternative *j* (where *J*=2, hypothetical treatment scenarios A and B) in choice set *s* (where *S*=18 pairs of scenarios per respondent) is given by:

$$[1] U_{isj}=V_{isj}+\varepsilon_{isj}=X'_{isj}\beta_{i}+\varepsilon_{isj}$$

where $V_{isj}$ is a systematic component of utility and $\varepsilon_{isj}$ is a stochastic component, *X_isj_* is a *K* x 1 vector of explanatory variables (*k* = 1 to *K* attribute levels) and *β_i_* is a conformable vector of coefficients (representing the effect of attribute levels on choice).

To allow for variation among men in their preferences for each treatment-related adverse effect and survival, a full random effects model was estimated. Each coefficients (*β)* was allowed to randomly vary over individuals but not over the repeated choices made by an individual by setting:

$[2] \beta_{ki}=\bar{\beta}_{k}+ \varpi_{ki}$ *k =* 1*,…,K*

where$\bar{\beta}_{k}$ is the mean parameter vector for the population and *ω_ki_* is the individual specific deviation from the mean. The *ω_ki_* are assumed to follow standard normal distributions, independent of each other and of the *ε_isj_*. This specification accounts for correlation among the 18 repeated choices per respondent. Conditional on *β_ki_*, and assuming the error terms *ε_isj_* to be identically and independently distributed (IID) as extreme value, the probability that individual *i* chooses *j* in scenario *s* is then given by:

Maximum simulated likelihood was implemented in STATA software, with 1,000 Halton draws [3]. The seven side effect attributes were dummy coded with the no side effect level in each attribute as the omitted (base) level. Survival was entered as a continuous variable with a linear and a quadratic term. As the quadratic term was not significant, it was dropped from the model. Survival range (life expectancy range) was also recoded as a continuous variable by using the difference between the two extreme (end) points for each level.

**Objective 1:** relative tolerability of the treatment-related adverse effects, was addressed by ranking the fixed effects coefficients from the random logit model. Those with the largest negative coefficients had the greatest negative impact on utility, and were therefore interpreted as least tolerable, and those with the smallest negative coefficients (having the least negative impact) or indeed positive coefficients (positive impact on utility), were interpreted as the most tolerable.

**Objective 2:** degree of variation among men in preferences, was quantified by the distribution of the random effect coefficients. Since these are assumed to be normally distributed, the majority (68%) lie within one standard deviation of the mean utility impact (fixed effect) coefficients. This range therefore represents the typical range of individual utility coefficients; as this is the most clinically relevant expression of preference heterogeneity, we present results in this way.

**Objective 3:** differences in preferences by treatment group were tested by estimating a model for each treatment group separately. Small sample sizes in these subgroups limited the estimable model to fixed effects models (ie, attribute coefficients were not allowed to vary over individuals). Further, since parameter estimates in each model are confounded with the respective error variances in discrete outcome models [4], the parameter estimates were not directly comparable across treatment groups, so rank ordering of parameter estimates was used to assess the similarity of preferences across the treatment groups.

**Objective 4:** effect of age on preferences for sexual function, was addressed by interacting age (dichotomized at 65) with severe erectile dysfunction and severe loss of libido. The likelihood ratio test was used to test the improvement in fit provided by this model as compared with the random coefficient logit model. While these models are nested, the hypothesis tests are non-standard because the parameter space is restricted under the alternative. The likelihood ratio test statistic therefore does not have the usual chi-square asymptotic distribution, and the appropriate critical value is somewhat smaller than usual chi-square value [5].

**Objective 5:** survival gains needed to justify persistent treatment-related adverse effects, were estimated as the value of changes in health utility associated with each level of each side effect expressed in terms of survival time. To do this, we adapted the concept of compensating variation from welfare economics [6].

Assuming that the systematic component of an individual’s utility, $V_{isj}$ (see [1] above), is defined over chronic health states (*h*) and survival duration (*T*), then for two mutually exclusive choices, the compensating variation (CV) is defined as:

$$[4] CV=\frac{1}{\mu}\left[\ln\left(1+e^{V^{0}}\right)-\left[\ln\left(1+e^{V^{1}}\right)\right]\right]$$

where: $\mu$ is the marginal utility of survival, ie the life expectancy coefficient for the 12 year level, *ß*_LE_; *V*^0^ is the systematic component of utility for a defined base case health state; and *V*^1^ is the systematic component of utility for the alternative health state.

Results in the main body of the paper are for a base case of mild loss of libido with no other problems (as this was the most prevalent health state for men who had initially been managed with active surveillance) and 12 year life expectancy.

This allows the ‘welfare impact’ (or in this case, wellbeing impact) of a change in health state, from *h*^0^ (health state three years after initial management with active surveillance) to *h*^1^ (health state three years after active treatment), with no change in survival time (*T*, held constant at 12 years), to be expressed as the difference in survival time (*CV*) that would satisfy the following equality:

$$[5]\text{ }V\left(h^{1},T+CV\right)=V\left(h^{0},T\right)=V^{0}$$

When treatments for localised prostate cancer cause chronic treatment-related adverse effects that lead to persistent decrements in quality of life, the survival ‘compensation’ (*CV* in [5]) is positive; extra survival is needed from a treatment to make its persistent treatment-related adverse effects worthwhile.

The CV estimates were based on the random coefficient logit model. In the estimation, 10,000 random draws from the reported distributions of the random parameters were used to simulate the CV using equation 4, implemented in STATA software. The mean of those 10,000 CVs was highly sensitive to outliers; hence the median was used as a more reliable summary measure. We replicated the process 1000 times to simulate a distribution of medians, and report the median and the 2.5 and 97.5 centiles, analogous to a bootstrap centile confidence interval.

Many men reported more than one treatment-related adverse effect persisting at three years. There were 3^7^ = 2187 unique health states described by the three levels of the seven adverse effects. It was not practicable to consider the survival gains needed for each one. Since the function used to calculate survival gains needed (expression 4 above) is not linear, the cumulative effect of multiple treatment-related adverse effects cannot be derived simply by summing the effects of the component effects. We therefore simulated the survival gains needed for the three most common treatment-related adverse effect profiles for each treatment group (see Table 4 in the manscript).

***Results not reported in the manuscript but relevant to calculations***

Additional life expectancy had a significant positive impact on utility (estimated coefficient (standard error) = 0.34 (0.024) per extra year of life, p<0.001), but range of life expectancy had no impact on utility (0.013 (0.0075), p=0.09).

***References***

1. Litwin, M.S., et al., *The UCLA Prostate Cancer Index: development, reliability, and validity of a health-related quality of life measure.* Medical Care, 1998. **36**(7): p. 1002-12.

2. McFadden, D. and K. Train, *Mixed MNL models for discrete response.* Journal of Applied Econometrics, 2000. **15**: p. 447-470.

3. Train, K., *Discrete choice methods with simulation*. 2003, New York: Cambridge University Press.

4. Swait, J. and J. Louviere, *The role of the scale parameter in the estimation and comparison of multinational logit models.* Journal of Marketing Research, 1993. **30**(3): p. 305-314.

5. Andrews, D.W.K., *Hypothesis testing with a restricted parameter space.* Journal of Econometrics, 1998. **84**: p. 155-199.

6. Lancsar, E. and E. Savage, *Deriving welfare measures from discrete choice experiments: inconsistency between current methods and random utility and welfare theory.* Health Economics, 2004. **13**(9): p. 901-907.
